# Evidence against the Involvement of Chronic Cerebrospinal Venous Abnormalities in Multiple Sclerosis. A Case-Control Study

**DOI:** 10.1371/journal.pone.0072495

**Published:** 2013-08-14

**Authors:** Ian W. Rodger, Dorothy Dilar, Janet Dwyer, John Bienenstock, Andu Coret, Judith Coret-Simon, Gary Foster, Arlene Franchetto, Slobodan Franic, Charles H. Goldsmith, David Koff, Norman B. Konyer, Mitchell Levine, Ellen McDonald, Michael D. Noseworthy, John Paulseth, Luciana Ribeiro, Mary Jane Sayles, Lehana Thabane

**Affiliations:** 1 Department of Medicine, McMaster University, Hamilton, Ontario, Canada; 2 Department of Clinical Epidemiology and Biostatistics, McMaster University, Hamilton, Ontario, Canada; 3 Department of Electrical and Computer Engineering, McMaster University, Hamilton, Ontario, Canada; 4 Department of Pathology and Molecular Medicine, McMaster University, Hamilton, Ontario, Canada; 5 Department of Neurology, McMaster University, Hamilton, Ontario, Canada; 6 Department of Radiology, McMaster University, Hamilton, Ontario, Canada; 7 Diagnostic Imaging, Hamilton Health Sciences, Hamilton, Ontario, Canada; 8 Clinical Research, Hamilton Health Sciences, Hamilton, Ontario, Canada; 9 Diagnostic Imaging, St Joseph's Healthcare, Hamilton, Ontario, Canada; 10 Biostatistics, St Joseph's Healthcare, Hamilton, Ontario, Canada; 11 Research Administration, St Joseph's Healthcare, Hamilton, Ontario, Canada; 12 Imaging Research Centre, St Joseph's Healthcare, Hamilton, Ontario, Canada; 13 Faculty of Health Sciences, Simon Fraser University, Vancouver, British Columbia, Canada; Cornell University, United States of America

## Abstract

**Objective:**

Multiple sclerosis (MS) is a chronic neurodegenerative disease of the CNS. Recently a controversial vascular hypothesis for MS, termed chronic cerebrospinal venous insufficiency (CCSVI), has been advanced. The objective of this study was to evaluate the relative prevalence of the venous abnormalities that define CCSVI.

**Methods:**

A case-control study was conducted in which 100 MS patients aged between 18–65 y meeting the revised McDonald criteria were randomly selected and stratified into one of four MS subtypes: relapsing/remitting, secondary progressive, primary progressive and benign. Control subjects (16–70 y) with no known history of MS or other neurological condition were matched with the MS cases. All cases and controls underwent ultrasound imaging of the veins of the neck plus the deep cerebral veins, and magnetic resonance imaging of the neck veins and brain. These procedures were performed on each participant on the same day.

**Results:**

On ultrasound we found no evidence of reflux, stenosis or blockage in the internal jugular veins (IJV) or vertebral veins (VV) in any study participant. Similarly, there was no evidence of either reflux or cessation of flow in the deep cerebral veins in any subject. Flow was detected in the IJV and VV in all study participants. Amongst 199 participants there was one MS subject who fulfilled the minimum two ultrasound criteria for CCSVI. Using MRI we found no significant differences in either the intra- or extra-cranial venous flow velocity or venous architecture between cases and controls.

**Conclusion:**

This case-control study provides compelling evidence against the involvement of CCSVI in multiple sclerosis.

## Introduction

Multiple sclerosis (MS) is a chronic (and often progressive), demyelinating, neurodegenerative, inflammatory disease of the central nervous system[1], [2]. However it has also been recognized that most MS plaques are situated around perivenular spaces. This observation led to the hypothesis that venous hemodynamic problems, including venous reflux, might be central to the underlying MS disease process [3]. The consequences of venous insufficiency in peripheral tissues are well established and include, amongst other things, microhemorrage and iron deposition [4]. It was characteristics such as these that led Zamboni and colleagues to hypothesize that MS was associated with abnormalities of the drainage of the cerebral venous system and that, along with vascular iron leakage, were involved in progression of the disease [5]–[9]. Zamboni and colleagues also coined the term chronic cerebrospinal venous insufficiency (CCSVI) to define the vascular condition present in MS patients in which anomalies of the main extracranial cerebrospinal venous architecture interfered with the normal venous drainage from the brain [5], [9]. The Zamboni hypothesis remains controversial [10]–[12]. In the past few years there have been numerous reports of studies examining the relationship between MS and CCSVI with highly variable results [13]–[24]. Furthermore, patients are electing to undergo interventions to open narrowed or blocked veins, a procedure not accepted or available in many medical environments. The primary objective of this study was to evaluate the relative prevalence of the five venous abnormalities that define CCSVI, using both Doppler ultrasound and magnetic resonance imaging (MRI) quantitative flow techniques, amongst four sub-groups of MS patients and the matched controls.

## Methods

### Subjects

Potential *cases* were screened from the MS clinic at McMaster University, Hamilton, Ontario. MS patients meeting the revised McDonald criteria for MS [25], [26] were stratified into one of four subtypes: (1) Relapsing/Remitting Multiple Sclerosis (RRMS) defined as patients having had two or more attacks, separated by at least 30 days. (2) Secondary Progressive Multiple Sclerosis (SPMS) defined as patients initially having a relapsing-remitting pattern but then showing gradual deterioration with increasing disability over a period of at least 12 months, independently of any clinically documented attacks. (3) Primary Progressive Multiple Sclerosis (PPMS) defined as patients with progressive disease right from the time of clinical onset, and who have been in this pattern for at least 12 months (with or without attacks superimposed). (4) Benign MS patients, defined for the purposes of this study as having a form of relapsing-remitting MS with disease duration of at least 5 years, attack rate less than 1 per year, all attacks being relatively mild and with a score on the Expanded Disability Status Scale (EDSS) <3.0 [27].

MS patients aged 18–65 years were randomly selected from a list of clinic patients and approached for recruitment into the study until 25 cases were enrolled into each of the four subtypes. A sample size of 25 per group (with 1∶1 matching with controls) permitted the detection of a doubling in the prevalence of CCSVI amongst MS patients, with 80% power and an alpha value of 5%.

*Control* subjects were required to be aged 16 to 70 years with no known history of MS or other neurological condition. Volunteer control subjects were screened and matched to the 100 cases on sex and age (within 5 y) and enrolled in the study. Controls could not be first or second degree relatives of the matched case. Exclusion criteria for cases and controls included: contraindications to MRI, significant claustrophobia, allergy to contrast agent (gadolinium or similar contrast) or known kidney disease, any known intra- or extra-cranial vascular malformation(s), history of deep vein thrombosis or pulmonary embolus, chronic venous insufficiency of the lower limbs, major head trauma, any prior neck surgery or central line insertion, or concurrent clinically important health problems.

All cases and controls had an ultrasound imaging procedure of the deep cerebral veins (DCV's) and the veins in the neck and magnetic resonance imaging of the neck veins and brain. For each participant the MRI and ultrasound procedures were performed on the same day. The vascular ultrasonographers were not blinded to the attribution of participants to either the MS patients' group or the control group but were blinded towards the MS subtypes of the patients. Radiologists reviewing either the ultrasound or MRI results were blinded to patient identity and to the results of the other imaging procedure.

### Cerebrospinal venous insufficiency criteria

The criteria for the presence of cerebrospinal venous insufficiency required two or more of the following features to be observed with ultrasound imaging (the “Zamboni rule”) [5], [9].

- reflux in the internal jugular veins (IJV) or the vertebral veins (VV) in sitting and supine posture.
- reflux or no flow in the deep cerebral veins (DCV).
- high-resolution B-mode evidence of proximal IJV anomalies or stenosis.
- flow not detectable by Doppler in the IJV's and/or VV's despite numerous deep inspirations in sitting and supine posture.
- reverted postural control of the main cerebral venous outflow pathways (IJV CSA supine-upright).

### Doppler ultrasound imaging procedures

Prior to the study two vascular ultrasonographers, each with more than 14 years experience, and one radiologist (with more than 30 years of ultrasonographic experience) traveled to Italy to be trained for one week in the Zamboni technique in the Department of Vascular Surgery at the Cliniche Nuove, Ferrara St Anna Archspedale, a clinical teaching facility affiliated with the University of Ferrara. Under the direct supervision of Dr Zamboni and his colleagues the ultrasound methods, techniques and criteria were presented and practiced.

Preceding the case-control study, ultrasound and MRI investigations were performed on 20 subjects (10 persons with MS and 10 healthy controls) to validate the techniques and to standardize the variables to be measured in the study. The results from these subjects are not included in the analysis.

The ultrasound examination was performed using a high resolution B-mode, pulsed-wave, color Doppler imaging ultrasound unit (Toshiba Aplio XG, Toshiba Medical Systems Corporation, Tochigi-ken, Japan). Extracranial vessels (IJVs and VVs) were evaluated with a 7–12 MHZ linear array transducer (PLT-704AT) and the deep intracranial veins (DCVs) were assessed with a 2.5 MHz sector probe (PST 25BT).

All scans were performed with minimal transducer pressure beginning with subjects in supine and then upright with the head in neutral position. High-resolution gray scale images of the IJV were assessed in longitudinal and transverse planes. The VV were only assessed in the longitudinal plane. Examinations commenced two minutes following a positional change. A number of deep breaths were taken by the subjects to allow for redistribution of blood within the venous system. The IJV assessment included three important levels; J1 at the level of the thoracic outlet, J2 at the level of the mid thyroid and J3 at the level of the carotid bifurcation.

Reflux or stenotic lesions of the IJV's and VV's were evaluated according to Zamboni criteria [5]–[7] for flow direction, flow velocity, cross sectional area (CSA) in relation to change in posture and anomalous morphology. According to our training in Italy, a stenosis was evaluated at the J1 level. An arbitrary peak velocity, increased pre-stenosis velocity and a decreased post-stenosis velocity were indicative of a stenosis. B mode imaging that demonstrated a CSA > = 50% reduction was also considered a stenosis. Our study was extended to assess flow direction, velocity and turbulence using pulse wave Doppler and sampling each vessel at a 60° angle along its entire length. With normal flow moving towards the heart, reflux was documented as a reversal of flow > 0.88 s while scanning in the longitudinal plane. Valsalva was never used with Zamboni's technique.

To be consistent with Zamboni's ultrasound technique, color flow analysis of the IJVs was also performed in the transverse plane. This technique produced inaccurate and ambiguous results. By changing the angle of insonation to the vessel or changing the pulse repetition frequency (PRF) we were able to demonstrate that flow direction, blockage and reflux could not be assessed in the transverse orientation.

Transcranial Doppler (TCD) sonography was performed with the subject in a supine position, using the standard transtemporal approach, optimizing with a low flow color setting, a low wall filter and a low PRF. As physiological intracranial venous flow is mono-directional we assessed for the presence or absence of reflux in at least one of the DCVs.

Using the trace calipers from the Toshiba measurements package, the cross sectional area (CSA) of the IJV was measured at J2 in both the supine and sitting positions. The CSA was measured during a short period of apnea following a normal exhalation. The change in CSA between supine and erect position was evaluated. The original analysis of this criterion by Zamboni [7] has been challenged as incorrect by Mayer *et al* [17] who proposed that the calculation should be ΔCSA supine - upright and not the other way around, an analysis with which we agree. Thus, we have adopted the Mayer *et al* proposal to analyse the data for this criterion.

### Quantitative magnetic resonance imaging venous flow

MRI was performed using a GE 3T Signa HD MRI system and an 8 channel neurovascular (NV) phased array RF coil (GE Healthcare, Milwaukee, WI). Initial cranial anatomical scans, both pre- and post-gadolinium contrast administration, were used to assess the brain parenchyma looking for unexpected abnormalities e.g., arteriovenous malformations (AVMs) that would have the potential to alter intra- and extra-cranial flow dynamics and hence disturb venous flow velocities. This step identified an AVM in one enrolled subject and the individual was excluded from the study analysis. Injection of gadolinium contrast was done during a coronal 3D MRV (magnetic resonance venogram) scan of the head and neck using an ATECO (auto-triggered elliptic centric-ordered) sequence (TE/TR = 2/7.8 ms, flip angle = 25°, 477Hz/pixel receiver bandwidth, 512×512matrix (no interpolation), 1.2 mm thick (0 mm skip), 32 cm FOV) [28]. Image acquisition was timed to occur during the venous phase of bolus passage. Contrast was injected at 4 cc/second into the left antecubital vein (Gadovist; 604 mg/mL gadobutrol, 0.2 mmol/kg; 20 gauge IV) using a Spectris Solaris power injector (Medrad, Warrendale, PA, USA).

Flow measurement was performed using a phase contrast (PC) flow sensitizing MRI pulse sequence, with axial-oblique acquisition, prescribed orthogonal to the vessels of interest (TE/TR = 4.14/42.15 ms, flip angle = 25°, 20 cm Field of View (FOV), 448×448 matrix, 4 mm thick, velocity encoding (venc) = 50 cm/s, and 30 points over the cardiac cycle). Three scans were performed, two through the IJV (both high and mid neck; supine position only) and one through the straight sinus (Figures S1a, S1b, S1c). Flow was quantified from PC phase images through integration of flow profiles throughout the cardiac cycle (taking into consideration both anterograde and retrograde flow) using the GE software “CV Flow” (GE Healthcare, Milwaukee, WI), run on an Advantage Windows workstation (v4.2). Using the modulus images, polygonal-shaped regions of interest (ROIs) were drawn on the vessel of interest and mean blood velocity (± 1 standard deviation) was plotted at each of the 30 points over a cardiac cycle (Figure S2). The CV Flow program is FDA and Health Canada approved and validated for measurement of physiological fluid flows. A comparison of flow in right and left vessels between cases and controls was conducted at each vascular level.

### Statistics

Baseline variables (both primary and secondary) are summarized using descriptive summary measures, expressed as mean (standard deviation) or median (minimum-maximum) for continuous variables and number (percent) for categorical variables.

Each of the five individual Zamboni criteria and the Zamboni rule were analyzed using exact conditional logistic regression. This method of analysis was chosen because so few criteria were satisfied (i.e., there were many zero counts). For continuous outcomes of flow the differences between case and control pairs were analyzed using the fixed-effects meta-analysis method to determine if there were differences between cases and controls. All MRI phase contrast flow measures were performed independently by two blinded analysts. Due to potential heterogeneity of ROI placement at the edges of blood vessels, we used the intra-class correlation coefficient [ICC] to assess consistency of measurements between them. We used the paired-t test to assess the MRI blood flow asymmetry (right IJV – left IJV) between MS cases and controls at the upper IJV and mid-neck IJV.

All statistical tests were performed using two-sided tests at the 0.05 level of significance. The Bonferroni method was used to adjust the level of significance for testing for secondary outcomes to keep the overall level at α = 0.05. For all models, the results are expressed as effect (or odds ratios for binary outcomes), standard errors, corresponding 95% confidence intervals and associated P-values when these statistics were estimable. In some instances they were not estimable due to the sparseness of the data. P-values were calculated to three decimal places and values less than 0.001 reported as <0.001. All analyses were performed using SAS V9.2 [29]. The analysis and reporting followed the STROBE (STrengthening the Reporting of OBservational studies in Epidemiology) guidelines [www.strobe-statement.org].

### Ethics statement

The protocol was approved by both the St Joseph's Healthcare Hamilton Research Ethics Board and the Hamilton Health Sciences/McMaster Faculty of Health Sciences Research Ethics Board before commencing. All study participants provided written informed consent after verbal discussion and reading of the hardcopy consent form. Both Research Ethics Boards approved the consent procedure.

## Results

The study was initiated in September 2010 and enrollment was completed in June 2011. Subjects were 74% female and a mean age of 48 years. The EDSS mean (SD) ranged from 1.94 (0.63) in the Mild group to 5.82 (1.95) in the PPMS group. The clinical and demographic data of the study participants are shown in Table 1.

**Table 1:** Clinical and Demographic Data.

Measurement	Benign MS (n = 25)	RRMS (n = 25)	SPMS (n = 25)	PPMS (n = 25)	Controls (n = 100)
**Age, yrs** (SD)	45.88 (10.43)	44.20 (9.21)	49.44 (9.20)	52.72 (7.93)	47.98 (9.02)
**Gender** female	76	76	84	60	74
**Duration of MS, yrs** mean (SD)	16.88 (10.36)	12.16 (7.50)	19.92 (10.47)	10.08 (6.84)	–
**EDSS** mean (SD)	1.94 (0.63)	3.82 (1.91)	5.72 (1.58)	5.82 (1.95)	–
**Annual Attack Rate** mean (min, max)	0.74 (0, 4)	1.67 (0, 8)	0.74 (0, 3)	0.14 (0, 2)	–
**Taking DMD's** n (%)	13 (54)	18 (78)	6 (24)	1 (4)	–
**Predominant Involvement** n (%)					
Cerebral	11 (46)	12 (50)	4 (16)	12 (48)	–
Spinal	2 (8)	3 (13)	7 (28)	2 (8)	–
Mixed	11 (46)	9 (38)	14 (56)	11 (44)	–

### Ultrasound

IJV and VV were detected 99.5% of the time in supine and 100% of the time in upright position. A DCV was documented in the supine position in 99.5% of the patients. There was no difference in the detection rates between both study groups.

#### CCSVI criteria I - Reflux in the IJV or VV in sitting and supine posture

We found no evidence of reflux using colour and duplex Doppler at any level bilaterally in either of the IJV or VV in any subject (Table 2). Reflux cannot be assessed in the transverse plane (Figure S3). Venous flow was spontaneous, phasic and caudal in both supine and upright positions (Figure S4). Reflux (>0.88 s) was never seen. In one control subject a vertebral vein was not visualized in the supine position.

**Table 2:** Zamboni Criteria.

Zamboni Criterion	Benign MS^1^ (n = 24)	RRMS (n = 25)	SPMS (n = 25)	PPMS (n = 25)	Controls (n = 100)	OR (95%CI)^2^	p-value^2^
Reflux in the internal jugular veins (IJV) or the vertebral veins (VV)	0	0	0	0	0	not estimable	>0.999
Reflux propagated upward to the deep cerebral veins (DCV)	0	0	0	0	0	not estimable	>0.999
High-resolution B-mode evidence of proximal IJV							
-stenosis	0	0	0	0	0	2.00 (0.18, 22.06)	0.571
-anomalies	0	1	0	1	1		
flow not detectable by Doppler in the IJV or VV even on inspiration	0	0	0	0	0	not estimable	>0.999
Change in IJV cross sectional area^3^	1	0	0	1	1	2.00 (0.18, 22.06)	0.571

1 data missing for one subject.

2 values based on exact conditional logistic regression (case vs control).

3 defined as ΔCSA (supine - upright) <0.

#### CCSVI criteria II - Reflux or no flow in the DCV

Flow in a DCV was assessed in the supine position. In one MS patient a DCV was not seen. There was no evidence of either reflux or cessation of flow in any of the study groups (Table 2). flow was mono-directional and did not change with either inspiration or expiration (Figure S5).

#### CCSVI criteria III - High Resolution B-mode evidence of IJV anomalies or stenosis

Gray scale assessment of the IJV displayed anomalies in 3 out of 199 subjects (1.5%, see Table 2). The B-mode anomalies appeared as malformed or irregular valve movements. In all subjects, flow patterns were not altered and the anomalies did not represent vascular narrowing.

There was no significant difference in maximum IJV velocities between matched cases and controls. After adjusting for the Bonferroni correction, none of these differences was statistically significant at any location (J1, J2, J3) or at either side (right or left).

#### CCSVI criteria IV - Flow not detectable by Doppler in the IJVs and/or VVs despite numerous inspirations in sitting and supine posture

Flow was detected in all patients with the exception of one control patient in whom one VV was seen in the upright position but not in supine. was displayed in both planes at J1, J2 and J3 by optimizing PRF, angle of insonation and color gains (Figure S6). There was no evidence of blockage found in either study group or at any of the levels measured (Table 2).

#### CCSVI criteria V - Reverted postural control of the main cerebral venous outflow pathways (IJV CSA supine-upright)

ΔCSA was calculated as supine - upright. A negative ΔCSA would reflect abnormal venous drainage in accordance with the Zamboni hypothesis that the supine CSA would be diminished in patients with MS. Two MS patients and one control subject demonstrated a negative ΔCSA (Table 2).

#### Zamboni criteria

The number of study participants who fulfilled each Zamboni criteria is shown in Table 2. No participants satisfied the first, second, or fourth criteria, three fulfilled the third criterion and three satisfied the fifth criterion. Exact conditional logistic regression analysis reveals no differences between cases and controls for any of the five criteria.

Amongst all participants only one PPMS patient fulfilled the minimum two of five Zamboni ultrasound criteria for CCSVI and none filled more than two (Table 3). Analysis of the Zamboni rule with exact conditional logistic regression reveals no difference between cases and controls (p = 0.991; Table 4). Due to the sparse nature of the data (i.e., infrequent positive criteria) we could not obtain a point estimate of the odds ratio and its corresponding 95% confidence interval.

**Table 3:** Zamboni Criteria.

# Zamboni Criteria Met	Control	Benign MS^1^	RRMS	SPMS	PPMS	Total
0	98	23	24	25	23	193
1	2	1	1	0	0	4
2	0	0	0	0	1	1
>2	0	0	0	0	0	0

1 data missing for one subject.

**Table 4:** Zamboni Rule.

Zamboni Rule	Controls	MS Patients^1^
<2 criteria met	100	98
≥2 criteria met	0	1

1 data missing for one subject test of Zamboni Rule using exact conditional logistic regression: p-value  = 0.991 OR and 95% CI not estimable.

### Magnetic resonance imaging

The between-reader reproducibility of MRI flow measurements was high—with ICC values varying from 0.81 to 0.94 for all segments.

**Figure 1:**
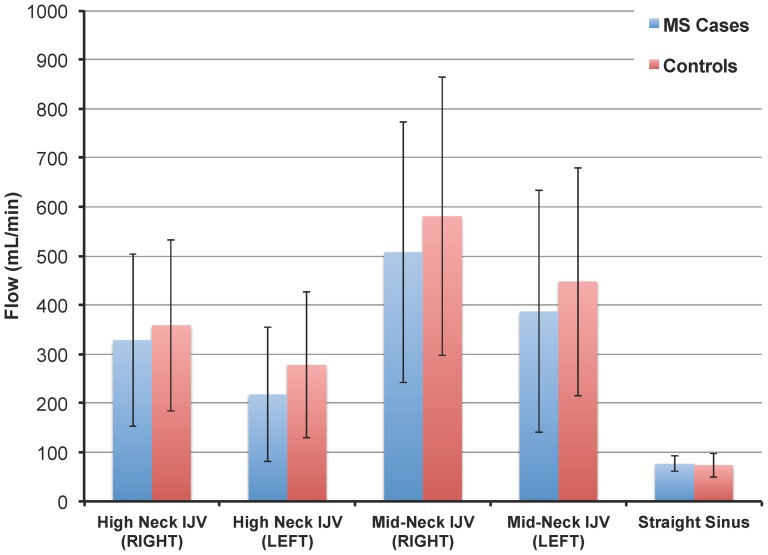
Comparison of absolute mean flow values (mL/min ± SD) between MS patients (blue) and their matched controls (red) measured in the right and left IJVs at the high neck and mid-neck regions as well as in the straight sinus. No significant differences were found between MS subjects and controls.

The comparison between absolute flow in the high neck and mid-neck IJVs as well as the straight sinus of all MS cases and control subjects is shown in Figure 1. There are no statistically significant differences between these flow values. A consistent finding was that flow in the right IJV was always higher than that in the left IJV. The differences in flow asymmetry (right IJV – left IJV) between MS cases and controls for the upper IJV and mid-neck IJV were not statistically significant (Table 5).

**Table 5:** Differences in flow asymmetry (right – left) between cases and controls for the upper and mid-neck IJV.

Flow asymmetry* (Right minus left)	Cases (n = 95)		Controls (n = 95)			
	Mean	SD	Mean	SD	t-test	p
**Upper IJV**	116.53	239.88	74.34	274.72	1.104	0.273
**Mid-neck IJV**	114.33	325.66	138.16	330.43	−0.528	0.599

IJV: Internal jugular veins; SD: standard deviation.

Examination of the extra- and intra-cranial venous anatomy using contrast MRV revealed no evidence of stenoses or other venous abnormalities between cases and controls.

## Discussion

In this case-control study, involving four types of MS patients with matched healthy controls, we found no evidence to support the hypothesis that CCSVI is associated with multiple sclerosis. Furthermore, given the relatively high age of the patient population in our study the data substantiate the view [30]–[32] that CCSVI is not a late secondary phenomenon of MS and is not associated with disability. Using Doppler ultrasound only one subject (with primary progressive MS) fulfilled the minimum criteria established by Zamboni et al [5], [9] for having CCSVI. However, at this juncture it is important to note that the “Zamboni criteria” as well as the proposed cut-offs are arbitrary in number and essentially an unproven hypothesis [33]. MRI quantitative flow assessment of extra- and intra-cranial venous blood flow revealed no clinically significant differences between the MS subjects or healthy matched controls. We did not find any evidence of CCSVI in any of the healthy subjects. Thus the data from this study do not support the existence of CCSVI.

Our data are consistent with several recent studies [12], [14], [17], [21], [24], [30], [32], [33]which found no evidence for CCSVI in MS patients using ultrasonographic techniques. The results from these studies are in marked contrast to those reported by Zamboni *et al* [5]–[7] who found that all MS patients exhibited CCSVI. In the ultrasound study by Zivadinov *et al* [18] the prevalence of CCSVI in MS subjects was 56%, an intermediate between the results reported by Zamboni and the results reported here and others [14], [17], [21], [24], [30], [32]. However, in the study by Zivadinov *et al* [18] 23% of the healthy control group were also reported to have CCSVI.

MRI techniques were not originally used to define CCSVI, but when they have been employed to examine extra-cranial venous flow in MS patients [13], [15], [16], [21] the data are not supportive of Zamboni's hypothesis. The results from our own MRI assessments are consistent with these other studies in that intra- and extra-cranial venous flow was not significantly different between cases and controls. The observation that right IJV flow was consistently higher than that in the left IJV is in accord with previously published data in both healthy [34] and MS subjects [21]. When one examines this flow asymmetry there is no significant difference in the differential between right minus left IJV flows for cases versus controls. Finally, on radiological examination of the contrast enhanced MRVs we found no evidence of structural abnormalities in the venous architecture of the extra-cranial vessels between cases and controls which is consistent with previous findings [12], [21], [35]. Collectively, these data serve to underscore the absence of any significant cerebrospinal venous abnormalities in MS subjects.

It is well recognized that ultrasonography is an operator-dependent technique and in studies utilizing ultrasound to evaluate CCSVI there are significant differences between the methodologies used and protocols described [12], [19], [20], [36]. In acknowledging this potential limitation, our vascular ultrasonographers and an ultrasound radiologist spent time with Zamboni's group in Ferrara, Italy to observe the technique that was used to define CCSVI.

Ultrasound is also subject to anatomical limitations. Intrathoracic vessels are obscured by bony anatomy and are not accessible. The deep cerebral veins that are accessible have limited fields of view. Despite this the sonographers were able to consistently demonstrate a DCV using the transtemporal approach. Furthermore, the extracranial veins respond to respiration, posture changes and breathing artifacts which demonstrate variable flow patterns making hemodynamic assessment limited and the evaluation of stenosis difficult. We are however confident in our ultrasound results since the MRI determinations that were performed on the same day as the ultrasonography are in accord that there were no significant differences in venous flow between the MS patients and their matched controls.

It thus appears that operator-induced venous abnormalities may account for the prior observation of CCSVI amongst patients with MS being investigated with ultrasound. We believe that the positive ultrasound findings in some previous studies [5]–[8], [18] reflect low specificity with the procedure, likely the product of operator-induced artifact. This would explain the almost bimodal heterogeneity for observing CCSVI demonstrated in a recent meta-analysis [19]. It is also unlikely that there was operator-related poor sensitivity for detecting CCSVI in the study that we conducted given that the MRI measures of flow in the study correlate well with ultrasound measurements of flow that were obtained in the same patient.

Another limitation of the study was our inability to blind the ultrasonographers regarding whether a subject was a MS patient or a control (e.g. MS patients may have required some assistance with mobility). In studies such as this one, an unblinding bias would be expected to increase the chance of observing an association. Given that no association was observed the lack of blinding appears to have been inconsequential. The radiologists reading either the ultrasound or MRI images were blinded to whether the participant was a MS patient or control subject, as well as to the results of the other imaging test. While viewing the MRI might reveal that a subject has MS (e.g. plaques being present) the fact that no association was observed between MRI flow abnormalities and MS patients in the study suggests that unblinding was not a problem.

## Conclusion

This case-control study provides compelling evidence against the involvement of CCSVI in MS.

## Supporting Information

Figure S1 **Flow was measured using a phase contrast (PC) flow encoding MRI sequence with slices chosen orthogonal to vessels of interest.** Both mid-neck (**fig. S1a**) and high-neck (**fig. S1b**) internal jugular vein were assessed bilaterally. In addition flow through the straight sinus (**fig. S1c**) was measured. Planes of PC acquisition are shown on post-contrast MRV scans.
(TIFF)

Figure S2 **Blood velocity (in cm/s) is shown though out a typical cardiac cycle in a venous structure.** Flow was quantified in 30 points over the cardiac cycle. A region of interest (ROI) was drawn on each venous structure using the GE software ‘CV flow’ and total flow (mL/min) in each was calculated. The plot shows mean blood velocity values measured at each of the 30 cardiac points (±1 standard deviation).
(TIFF)

Figure S3 **Colour Doppler transverse images of the IJV and CCA at the level of the thyroid.** Cranial and caudal angulation of the probe in the transverse orientation creates opposite colour representation of the IJV and should not be interpreted as reflux.
(TIF)

Figure S4 **Longitudinal spectral Doppler of IJV in supine position at J1 level.**
(TIF)

Figure S5 **Transcranial axial colour Doppler (TCD).** Transtemporal approach. Colour Doppler imaging and Doppler spectrum. No change in flow direction with inspiration (I) and expiration (E).
(TIF)

Figure S6 **Colour Doppler transverse image of the IJV and CCA at J3 level in sitting position.** Absence of flow when the pulse repetitions frequency(PRF) sensitivity is set too low and presence of flow when PRF is increased to appropriate sensitivity level.
(TIF)
