# Supplementary figures and images for: Evidence against the Involvement of Chronic Cerebrospinal Venous Abnormalities in Multiple Sclerosis. A Case-Control Study
Source: PLoS One. 2013 Aug 14;8(8):e72495. doi: 10.1371/journal.pone.0072495 (PMC3743778; doi:10.1371/journal.pone.0072495)

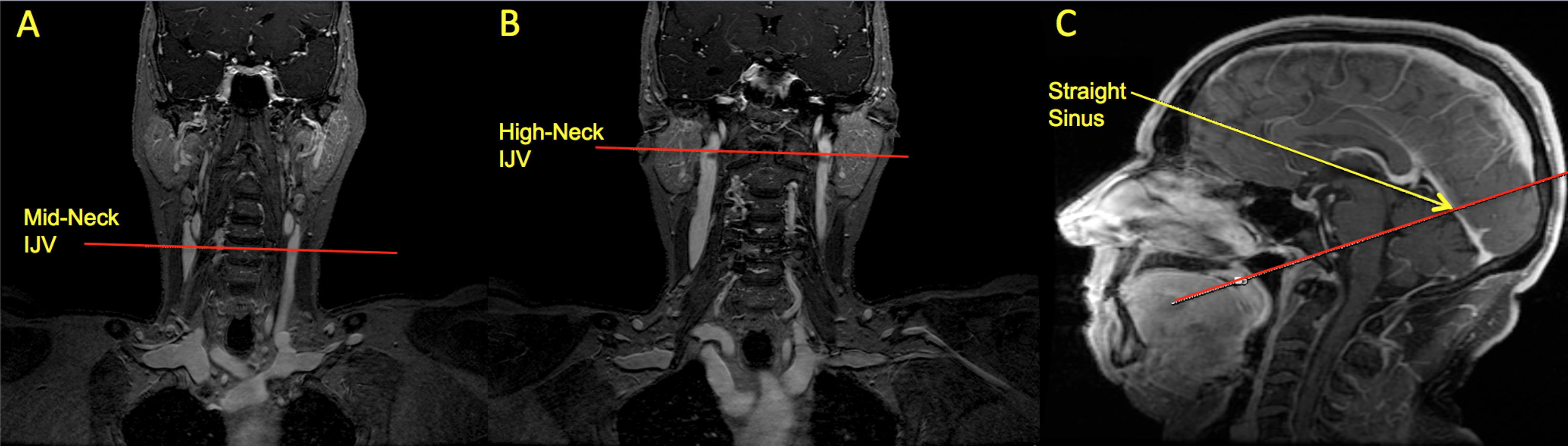

Supplement: Figure S1 — Flow was measured using a phase contrast (PC) flow encoding MRI sequence with slices chosen orthogonal to vessels of interest. Both mid-neck (fig. S1a) and high-neck (fig. S1b) internal jugular vein were assessed bilaterally. In addition flow through the straight sinus (fig. S1c) was measured. Planes of PC acquisition are shown on post-contrast MRV scans. (TIFF) [file pone.0072495.s001.tif]

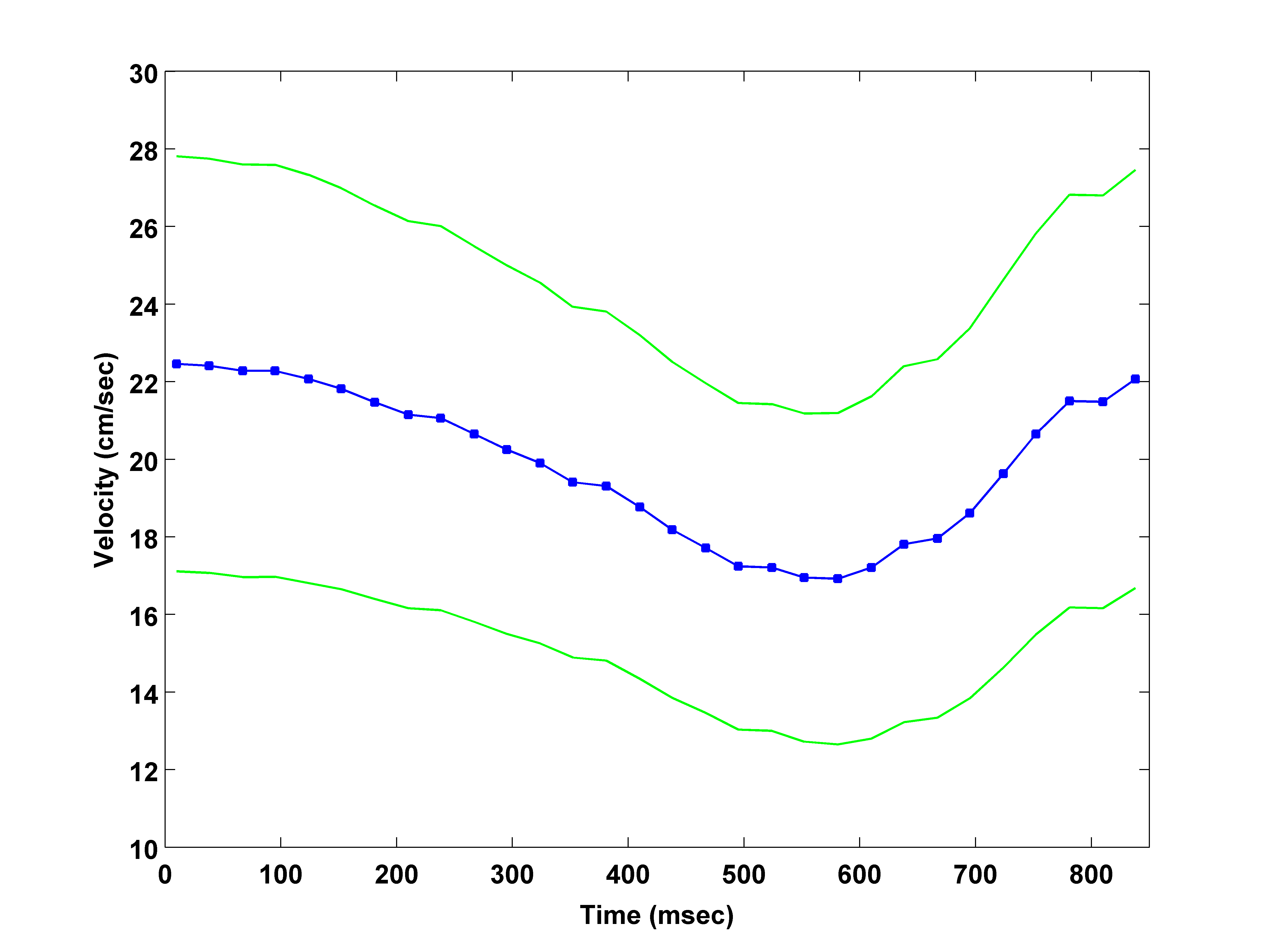

Supplement: Figure S2 — Blood velocity (in cm/s) is shown though out a typical cardiac cycle in a venous structure. Flow was quantified in 30 points over the cardiac cycle. A region of interest (ROI) was drawn on each venous structure using the GE software ‘CV flow’ and total flow (mL/min) in each was calculated. The plot shows mean blood velocity values measured at each of the 30 cardiac points (±1 standard deviation). (TIFF) [file pone.0072495.s002.tif]

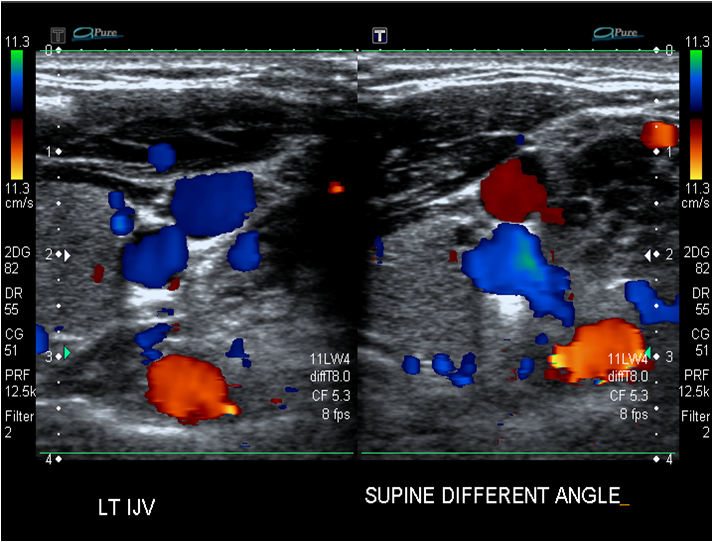

Supplement: Figure S3 — Colour Doppler transverse images of the IJV and CCA at the level of the thyroid. Cranial and caudal angulation of the probe in the transverse orientation creates opposite colour representation of the IJV and should not be interpreted as reflux. (TIF) [file pone.0072495.s003.tif]

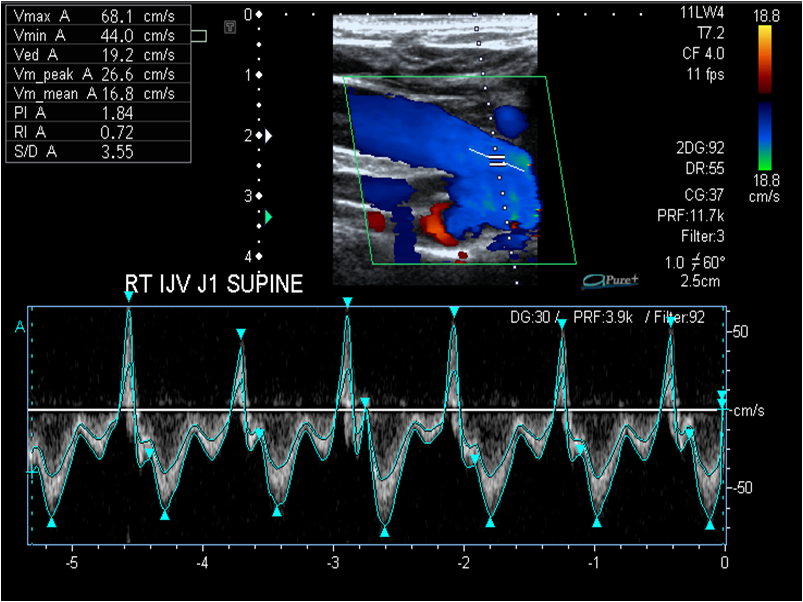

Supplement: Figure S4 — Longitudinal spectral Doppler of IJV in supine position at J1 level. (TIF) [file pone.0072495.s004.tif]

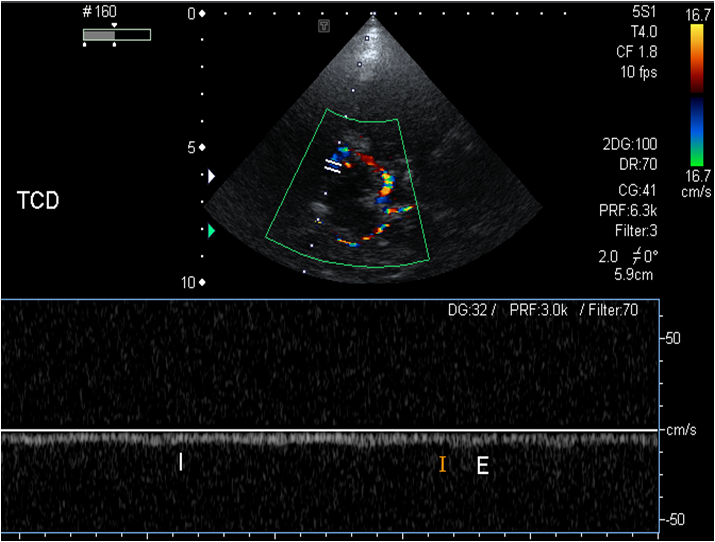

Supplement: Figure S5 — Transcranial axial colour Doppler (TCD). Transtemporal approach. Colour Doppler imaging and Doppler spectrum. No change in flow direction with inspiration (I) and expiration (E). (TIF) [file pone.0072495.s005.tif]

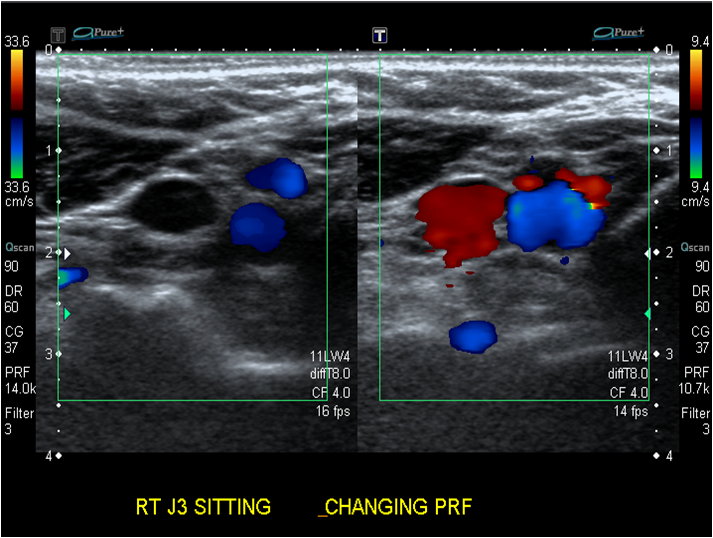

Supplement: Figure S6 — Colour Doppler transverse image of the IJV and CCA at J3 level in sitting position. Absence of flow when the pulse repetitions frequency(PRF) sensitivity is set too low and presence of flow when PRF is increased to appropriate sensitivity level. (TIF) [file pone.0072495.s006.tif]
